# Genetic diversity and structure of *Capsicum annuum* as revealed by start codon targeted and directed amplified minisatellite DNA markers

**DOI:** 10.1186/s41065-019-0108-6

**Published:** 2019-10-16

**Authors:** David O. Igwe, Celestine A. Afiukwa, George Acquaah, George N. Ude

**Affiliations:** 1 Department of Biotechnology, Faculty of Science, Ebonyi State University, Abakaliki, 053 Nigeria; 2 Biotechnology and Research Development Centre, Ebonyi State University, Abakaliki, Ebonyi State 053 Nigeria; 3 Department of Natural Sciences, Bowie State University, 14000 Jericho Park Road, Bowie, Maryland 20715 USA

**Keywords:** Cetyltrimethylammonium bromide, Germplasm, Shannon’s information index, Percentage polymorphic loci, Nei’s genetic diversity, Estimate of gene flow, *Capsicum annuum*

## Abstract

**Background:**

Identification of high resolving DNA-based markers is of paramount importance to unlock the potential of genetic diversity and selection of unique accessions of *Capsicum annuum* L., within Cross River and Ebonyi States of Nigeria, for breeding and conservation. Therefore, we comparatively explored the effectiveness of start codon targeted (SCoT) and directed amplified minisatellite DNA (DAMD) markers for diversity analysis of the accessions. Fifteen accessions were collected for DNA extraction and amplifications with the markers.

**Results:**

Dendrograms from SCoT and DAMD categorized the accessions into five and three genetic groups, respectively, while the principal component analysis identified five genetic clusters, each from the markers. The average values of allele, gene diversity and polymorphic information content detected with SCoT and DAMD demonstrate that the two markers were effective and efficient, especially, SCoT in genetic diversity study of the accessions of pepper. Number of polymorphic loci (NPL) and percentage polymorphic loci (PPL) from SCoT (NPL = 64, PPL = 80.00–95.73%) and DAMD (NPL = 56, PPL = 53.33–86.67%) were high, but higher in SCoT markers. Other effective genetic parameters (effective number of alleles, Nei’s genetic diversity and Shannon’s information indices) identified with the two marker systems elucidated the allelic richness, rich genetic diversity within the populations and informative nature of the markers, especially SCoT. The intraspecific genetic diversity, interspecific genetic diversity, and coefficient of differentiation obtained with SCoT and DAMD further exposed the genetic structure with more genetic divergence within than among the populations of the accessions. Estimate of gene flow from the SCoT markers was 3.8375 and 0.6.2042 for the DAMD markers. The estimate of gene flow values from the markers indicated extensiveness with SCoT (Nm = 3.8375) and extremely extensive with DAMD (Nm = 6.2042) among the populations.

**Conclusion:**

This study shows that SCoT markers may be more useful and informative than DAMD in measuring genetic diversity and differentiation of the accessions of the genus *Capsicum*. Genetic parameters obtained with SCoT showed that the accessions from Cross River were more genetically diverse than the ones from Ebonyi State. Therefore, SCoT may be a preferred marker in evaluating genetic diversity for improvement and conservation of this spicy crop, *C. capsicum*.

## Background

Pepper (*Capsicum* spp.), belonging to family Solanaceae and sub-family Solanoideae is grown worldwide for its vital uses as vegetables, spices, ornaments, medicines, lachrymatories, vitamins A and C [1–7]. It was discovered as early as 6000 years ago and it is regarded as the first spice utilized by human beings [3, 8]. The ancestral sources are Central and Southern America hinged in Bolivia, while Brazil is the center for genetic diversity of this important spice [9–12]. Thirty-eight species of *Capsicum* have been reported out of which *Capsicum annuum* L., *C. frutescens* L.*, C. Chinense* Jacq., *C. baccatum* L. (aji), and *C. pubescens* Ruiz and Pay were considered to have been domesticated and maintained through more than five independent occurrences [9, 13, 14]. Some of these species were considered to have undergone a process of an independent domestication in at least two zones of the New World including Mesoamerica (*C. annum* and *C. frutescens*) and South America (*C. chinese*, *C. baccatum* and *C. pubescens*) [9, 15]. The cultivated species (*C. annuum*, *C. frutenscens*, *C. chinese*, *C. pubescens* and *C. baccatum* var. *pendulum*) are of great economic importance and they possess good attributes that can be maintained through domestication [8, 16, 17]. However, continuous selection during domestication brought about emergence of novel lines with phenotypic traits including larger, non-pungent fruit with greater shape variation and tremendous increases in fruit mass [18, 19]. Breeding programs targeting crop improvement are accelerated by the availability of well-characterized genetically diverse germplasm of pepper accessions and this is obtainable through the application of informative molecular markers to complement the roles of a morphology-based identification process.

Many marker systems of different categories including amplified fragment length polymorphisms (AFLP), simple sequence repeat (SSR), random amplified polymorphic DNA (RAPD), isozymes, inter-simple sequence repeat (ISSR), restriction fragment length polymorphism (RFLP), gene-based markers of start codon targeted and expressed sequence tag-simple sequence repeat (SCoT and EST-SSR), and single nucleotide polymorphism (SNP) have been utilized in studying and characterizing genetic loci linked to diversity, relationships, variations, parental selection, cultivar identity, phenotypic traits, purity, population studies, and resistance to diseases in *Capsicum* species [20–42]. The basic information derivable from genetic variability of these potential markers could provide useful information that will assist plant breeders to manage and improve germplasm [23]. Use of microsatellite markers that are polymorphic, multi-allelic, reproducible and widely distributed in the pepper accessions could facilitate the selection of traits of interest and potential breeding materials for introgression through molecular marker-assisted breeding and germplasm conservation [23, 43, 44]. Functional gene-based marker such as SCoT, has been applied in other crops (*Elymus sibiricus)* [45]; (*Boehmeria nivea* L. Gaudich) [46]; and (*Vigna unguiculata* L.) [22]. Also another potential marker, directed amplified minisatellite DNA (DAMD), has also been useful in other crops including *Flavoparmelia caperata* [5]; *Musa acuminata* colla [47]; and Citrus [48].

Start codon targeted markers that are derivable from transcribed regions of the genome can be potentially useful for various applications in plant genotyping since they are capable of exposing polymorphisms that might be directly related to gene functions. These molecular markers were initially described, based on the observation that the short conserved regions of plant genes are surrounded by the ATG translation start codon [49, 50]. The utility of SCoT was advocated due to their inherent features such as reproducibility, possibility of getting co-dominance during amplification, accuracy in investigation of genetic relatedness and genetic diversity of plants [50–54]. Also, DAMD markers, believed to be tandemly repeated units of genomes are satellite marker systems that have become valuable and of great importance in identification of genotypes following discovery [55]. They exhibit many variations as inversional mutations that result in their orientation and distribution on both strands of the DNA sequences [56, 57]. Till now, SCoT and DAMD marker techniques have not been comparatively applied in the assessment of genetic diversity of pepper. Therefore, the objective of this study is to compare the efficacies of SCoT and DAMD markers in assessing genetic diversity among the accessions of *C. annum* from Cross River and Ebonyi States of Nigeria.

## Methods

### Plant materials

Fifteen accessions of *C. annuum* were collected from Cross River and Ebonyi States of Nigeria (Additional file 1: Table S1). One accession was purposefully obtained from each of the fifteen locations across Local Government Areas (LGAs) in Cross River and Ebonyi States to assess the efficiency of the two DNA-based molecular markers in studying the genetic diversity of *C. annuum*.

### DNA extraction

DNA extraction of the accessions was carried out using a modified Cetyltrimethylammonium bromide (CTAB) method [58]. Briefly, a young leaf of *C. annuum* was collected and weighed between 100 and 200 mg. It was thoroughly ground with 500 μL of the extraction buffer (EB: 2% CTAB, 100 mM Tris-HCl, 20 mM EDTA, 1.4 M NaCl and 0.2% β-mercaptoethanol) and later made up with additional 200 μL of the EB before transferring to a sterile 1.5 ml microcentrifuge tube for homogenous mixture. It was incubated at 65 °C for 15 min followed by addition of equal volume of phenol, chloroform and iso-amyl alcohol in the ratio of 25:24:1. It was mixed and centrifuged at 14,000 rpm (rpm) for 15 min after which 500 μL of the supernatant was transferred to a new sterile 1.5 ml microcentrifuge tube. It was precipitated by adding 400 μL of cold-isopropanol, followed by gentle inversion prior to incubation at -20 °C for overnight. It was centrifuged at 12,000 rpm to sediment the DNA prior to washing with 600 μL of 70% ethanol. It was subsequently air-dried and eluted with 100 μL of sterile water for polymerase chain reaction (PCR) set up.

### Polymerase chain reaction and agarose gel electrophoresis

A total of 10 SCoT [47] and 10 [59] DAMD primers were used in this study. Polymerase chain reaction amplification was performed in volume of 25 μL consisting of 2.0 μL 100 ng DNA, 2.5 μL of 10 x Buffer (Bioline), 1.5 μL of 50 mM MgCl_2_ (Bioline), 2.0 μL of 2.5 mM dNTPs (Bioline), and 0.2 μL 500 U Taq DNA polymerase (Bioline), 1.0 μL of 10 μM each of the SCoT and DAMD primers (Additional file 2: Table S2) and 15.80 μL of 500 ml diethylpyrocarbonate (DEPC)-treated water (Invitrogen Corporation, USA). The PCR cycling profile used for the reaction consisted of an initial step at 95 °C for 5 min., 40 cycles of 94 °C for 30s, 55-65 °C for 35 s, 72 °C for 1 min, and a 10-min final extension at 72 °C. Eight (8) μL of the PCR products were electrophoresed in a 1.5% agarose gel containing 0.5 mg/ml ethidium bromide and photographed on Transilluminator UV light (Fotodyne Incorporated, Analyst Express, USA).

### Data analysis

Data matrix of SCoT and DAMD marker profiles for fragments of similar molecular weights from each amplicon or individual were scored as 1 (presence of alleles) and 0 (absence of alleles). The data achieved from the scoring of reproducible bands from SCoT and DAMD amplicons after the second repeated experiments were used for dendrogram reconstruction using Unweighted Pair Group Method with Arithmetic Mean (UPGMA) and dissimilarity index in Jaccard’s option [60]. The analysis was conducted using Numerical Taxonomy and Multivariate Analysis System (NTSYSpc) software version 2.02. Furthermore, the genetic diversity, allele frequency and polymorphic information content (PIC) were computed using PowerMarker (Version 3.25). DARwin version 5 was used for principal component analysis (PCA), while effective number of alleles (Ne), Nei’s genetic diversity (H), and Shannon’s information index (I) [61] and population structure analyses including total genetic diversity (intraspecific diversity, Ht), gene diversity within the population (interspecific diversity, Hs), coefficient of differentiation (G_ST_) and estimate of gene flow (Nm) of the accessions were done using POPGENE software version 1.32.

## Results

### Genetic diversity of accessions of *Capsicum annuum* as identified by start codon targeted markers

**Fig. 1:**
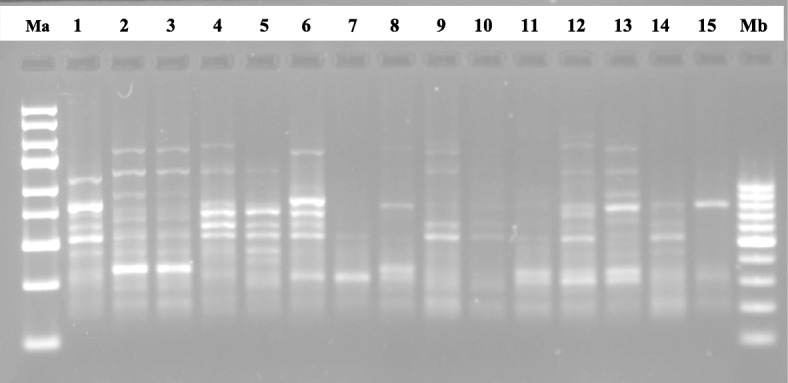
Fifteen *Capsicum annuum* DNA samples amplified with SCoT13 marker. Ma = 1 kb DNA ladder; Mb = 100 bp DNA ladder; 1–8 = *C. annuum* accessions from different locations of Cross River State, Nigeria; and 9–15 = *C. annuum* accessions from different locations of Ebonyi State, Nigeria

**Fig. 2:**
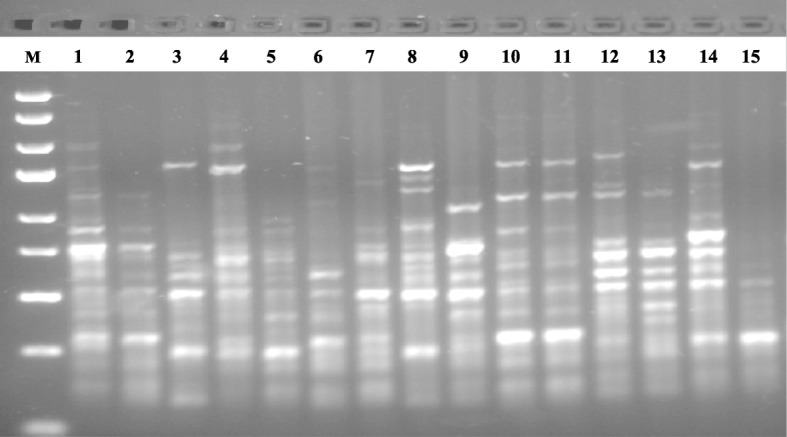
Fifteen *Capsicum annuum* DNA samples amplified with SCoT20 marker. M = 1 kb DNA ladder; 1–8 = *C. annuum* accessions from different locations of Cross River State, Nigeria; and 9–15 = *C. annuum* accessions from different locations of Ebonyi State, Nigeria

**Fig. 3:**
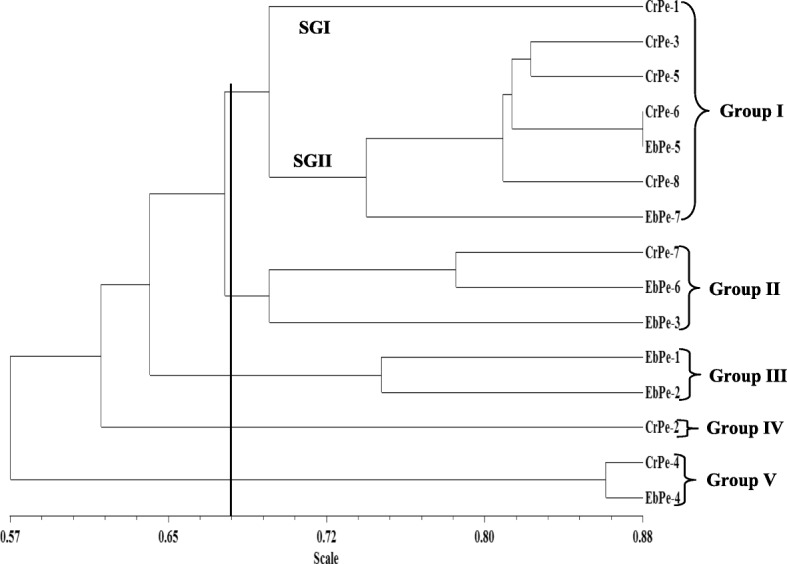
Dendrogram of 15 accessions of *Capsicum annuum* amplified with start codon targeted markers

To discern the level of genetic diversity in 15 accessions of *C. annuum* studied, a total of 10 SCoT primers were used for the study. Out of the 10 primers used, five (5) of them produced reproducible bands that were used in the analyses as exemplified in the gel images of SCoT13 and SCoT20 markers (Figs. 1 and 2). A dendrogram of the 15 accessions using UPGMA procedure clustered the accessions into five major groups at a genetic distance threshold of 0.666 (Fig. 3). Group I was subdivided into subgroups I and II denoted as SGI and SGII, respectively. The SGI clustered CrPe-1, while SGII had CrPe-3, CrPe-5, CrPe-6, EbPe-5, CrPe-8 and EbPe-7. The accession EbPe-7 was the most genetically isolated one in the group followed by CrPe-8. The accession (CrPe) in SGI was collected from Cross River, while those in SGII were from both Cross River and Ebonyi (EbPe) States. Group II had CrPe-7, EbPe-6 and EbPe-3 and they were from Cross River and Ebonyi States. Group III clustered together accessions EbPe-1 and EbPe-2 from Ebonyi. Group IV had only CrPe-2 accession that was collected from Cross River State. Group VI contained CrPe-4 and EbPe-4 accessions that were collected from Cross River and Ebonyi States, respectively. Principal component analysis of the generated amplicons resulted into five clusters (Additional file 3: Fig. 1). The clustering of each is a representative of unique accession or accessions of *C. annuum*.

The five SCoT markers amplified a total of 57 alleles (Table 1). The amplified alleles from each primer ranged from 7 to 14, with a mean of 12.000. Polymorphic information content values ranged from 0.7735–0.9193, with a mean value of 0.8709. The SCoT markers including SCoT13, SCoT28, SCoT20, SCoT24 and SCoT16 were found to be polymorphic. The genetic diversity ranged from 0.7911–0.9244, with a mean of 0.8815, while major allele frequency spanned from 0.1333–0.4000, with a mean of 0.2000. The identified allelic counts and frequencies from each of the SCoT loci ranged from 1 to 6 and 0.0667–0.4000, respectively (Additional file 4: Table S3).

**Table 1:** Allele frequency, number of alleles, genetic diversity and polymorphic information content of start codon targeted

Marker	Major allele frequency	No of Allele	Gene diversity	PIC
SCoT13	0.1333	14.0000	0.9244	0.9193
SCoT28	0.1333	13.0000	0.9156	0.9093
SCoT20	0.1333	14.0000	0.9244	0.9193
SCoT24	0.3333	7.0000	0.8000	0.7746
SCoT16	0.4000	9.0000	0.7911	0.7735
Mean	0.2000	12.0000	0.8815	0.8709

*PIC* Polymorphic information content

The genetic diversity in CrPe-2 was identified to be highest among the accessions, with effective number of alleles (Ne), Nei’s genetic diversity (H), and Shannon’s information index (I) values of 1.9996, 0.4999 and 0.6931, respectively (Table 2). On the other hand, the genetic diversity in CrPe-4 was the lowest, with Ne, H, and I values of 1.0270, 0.0263 and 0.0708, respectively. The genetic diversity indices of these accessions were ranked in an ascending order as CrPe-2 > (CrPe-8, EbPe-1) > CrPe-6 > CrPe-3 > (CrPe-5, EbPe-5) > CrPe-7 > CrPe-1 > (EbPe-2, EbPe-3) > EbPe-4 > CrPe-4. Effective number of alleles, H and I values spanned from 1.4149 ± 0.2594–1.8026 ± 0.3338, 0.2702 ± 1379–0.4160 ± 0.1701 and 0.4218 ± 0.1962–0.5831 ± 0.2376, respectively. The mean values of Ne, H and I obtained from the accessions collected within Cross River State were 1.7373, 0.4011 and 0.5747, respectively, while those from Ebonyi had 1.6568, 0.3850 and 0.5685, respectively. The overall mean values of Ne, H and I detected in the accessions from the two States using SCoT were 1.6971, 0.5936 and 0.8590, respectively.

**Table 2:** Analysis of genetic diversity of 15 accessions of *Capsicum annuum* species using start codon targeted

Pepper Accession	Effective no of alleles, Ne	Nei’s genetic diversity, H	Shannon’s information index, I
CrPe-1	1.7082	0.4146	0.6051
CrPe-2	1.9996	0.4999	0.6931
CrPe-3	1.8281	0.4530	0.6454
CrPe-4	1.0270	0.0263	0.0708
CrPe-5	1.7705	0.4352	0.6269
CrPe-6	1.9023	0.4743	0.6672
CrPe-7	1.7399	0.4252	0.6164
CrPe-8	1.9231	0.4800	0.6730
Sub-mean	1.7373	0.4011	0.5747
EbPe-1	1.9231	0.4800	0.6730
EbPe-2	1.6423	0.3911	0.5799
EbPe-3	1.6423	0.3911	0.5799
EbPe-4	1.2037	0.1692	0.3102
EbPe-5	1.7705	0.4352	0.6269
EbPe-6	1.7399	0.4252	0.6164
EbPe-7	1.6756	0.4032	0.5930
Sub-mean	1.6568	0.3850	0.5685
Overall mean	1.6971	0.5936	0.8590

*CrPe* Cross River State pepper accessions and *EbPe* from Ebonyi State

The genetic variation in pepper accessions from Cross River assessed using SCoT markers, revealed that the mean values of total gene diversity (Ht), gene diversity within population (Hs), coefficient of gene differentiation (G_ST_) and level of gene flow (Nm) were 0.4011, 0.3729, 0.0685 and 9.2368, respectively (Table 3). Values of Ht, Hs, G_ST_ and Nm recorded in the accessions from Ebonyi were 0.3850, 0.3200, 0.1918 and 2.8444, respectively. The overall values of Ht, Hs, G_ST_ and Nm obtained from the entire populations were 0.3936, 0.3482, 0.1152 and 3.8375, respectively. The G_ST_ value recorded 0.1153 indicating about 12% of the total genetic divergence among the populations and the remaining 88% within the populations. Across the SCoT markers, the numbers of polymorphic loci (NPL) and percentage polymorphic loci (PPL) were 12–13 and 80.00–95.73%, respectively (Additional file 5: Table S4). Effective number of alleles, H and I values obtained were 1.4149 ± 0.2594–1.8026 ± 0.3338, 0.1702 ± 0.1379–0.4160 ± 0.1701 and 0.4218 ± 0.1962–0.5831 ± 0.2376, respectively.

**Table 3:** Genetic diversity and differentiation in the accessions of *Capsicum annuum* using start codon targeted

Pepper Accession	Ht	Hs	G_ST_	Nm
CrPe-1	0.4146	0.3840	0.0738	6.2791
CrPe-2	0.4999	0.4907	0.0185	26.5385
CrPe-3	0.4530	0.4089	0.0973	4.6371
CrPe-4	0.0263	0.0249	0.0541	8.7500
CrPe-5	0.4352	0.4124	0.0523	9.0625
CrPe-6	0.4743	0.4409	0.0705	6.5957
CrPe-7	0.4252	0.4018	0.0552	8.5606
CrPe-8	0.4800	0.4196	0.1259	3.4706
Sub-mean	0.4011	0.3729	0.0685	9.2368
EbPe-1	0.4800	0.4338	0.0963	4.6923
EbPe-2	0.3911	0.2916	0.2545	1.4643
EbPe-3	0.3911	0.3520	0.1000	4.5000
EbPe-4	0.1692	0.0996	0.4118	0.7143
EbPe-5	0.4352	0.3804	0.1258	3.4740
EbPe-6	0.4252	0.3698	0.1304	3.3333
EbPe-7	0.4032	0.3129	0.2240	1.7323
Sub-mean	0.3850	0.3200	0.1918	2.8444
Overall mean	0.3936	0.3482	0.1153	3.8375
St. Dev	0.0163	0.0161		

*Ht* total gene diversity, *Hs* gene diversity within population, *G*_*ST*_ coefficient of gene differentiation and *Nm* estimate of gene flow from G_ST_ or Gcs. E.g., Nm = 0.5(1 - G_ST_)/G_ST_, *CrPe* population of pepper from Cross River State and *EbPe* from Ebonyi State

### Genetic diversity of accessions of *Capsicum annuum* as identified by directed amplified minisatellite DNA markers

**Fig. 4:**
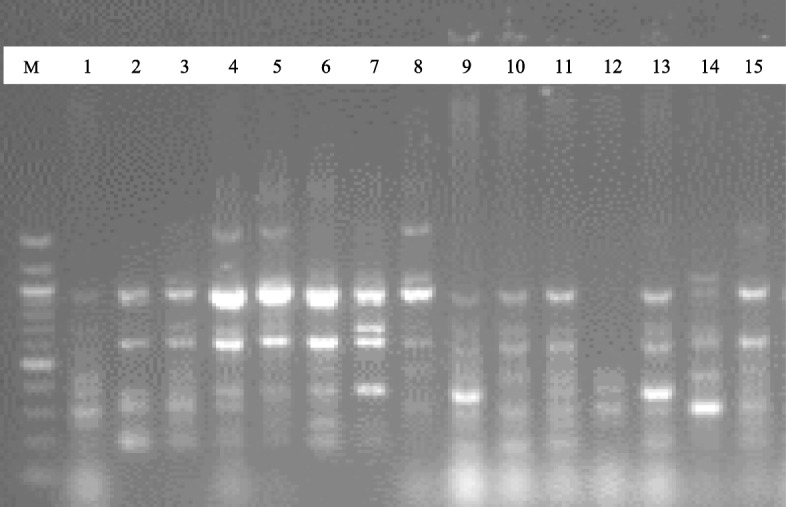
Fifteen *Capsicum annuum* DNA samples amplified with DAMD25 marker. M = 100 bp DNA ladder; 1–8 = *C. annuum* accessions from different locations of Cross River State, Nigeria; and 9–15 = *C. annuum* accessions from Ebonyi State, Nigeria

**Fig. 5:**
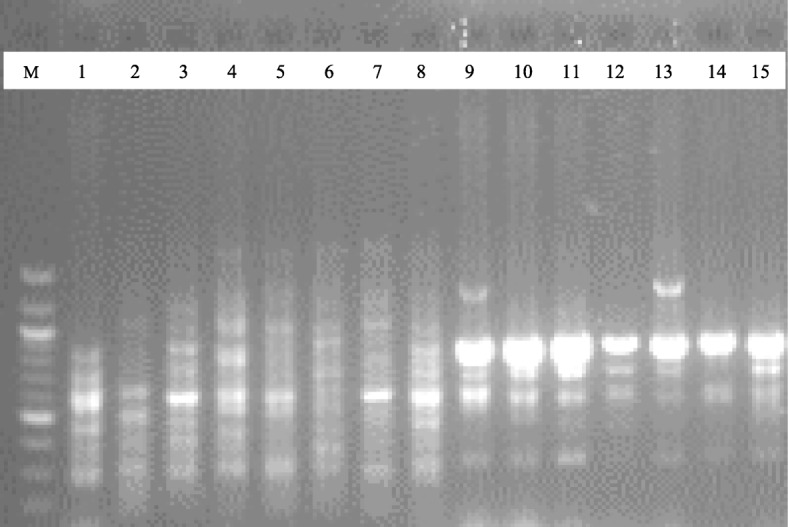
Fifteen *Capsicum annuum* DNA samples amplified with DAMD16 marker. M = 100 bp DNA ladder; 1–8 = *C. annuum* accessions from different locations of Cross River State, Nigeria; and 9–15 = *C. annuum* accessions from Ebonyi State, Nigeria

**Fig. 6:**
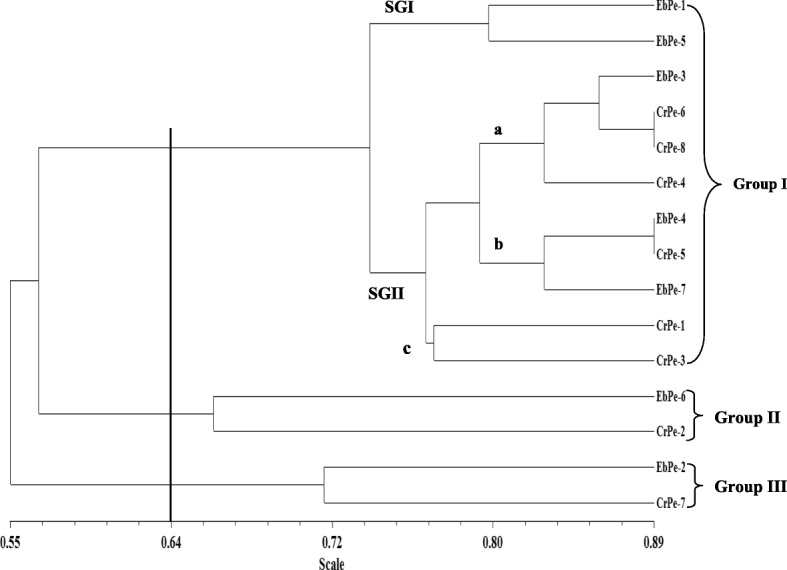
Dendrogram of 15 accessions of *Capsicum annuum* amplified with directed amplified minisatellite DNA markers

To ascertain the level of genetic diversity in 15 accessions of *C. annuum*, a total of 10 DAMD primers were used for the accessions. Out of the 10 primers used, five (5) reproducible primers obtained were used in the analyses as demonstrated in the gel images of DAMD25 and DAMD16 primers (Figs. 4 and 5). A dendrogram of the 15 accessions using UPGMA procedure clustered them into three major groups at a genetic distance threshold of 0.6400 (Fig. 6). Group I was subdivided into subgroups I and II denoted as SGI and SGII, respectively. The SGI consisted of EbPe-1 and EbPe-5 accessions from Ebonyi State, while SGII was further subdivided into a (EbPe-3, CrPe-6, CrPe-8 and CrPe-4), b (EbPe-4, CrPe-5 and EbPe-7) and c (CrPe-1 and CrPe-3). Group II had EbPe-6 and CrPe-2, while group III clustered accessions EbPe-2 and CrPe-7. Principal component analysis of yielded five clusters (Additional file 6: Fig. 2). Each cluster contained an accession or accessions of *C. annuum* based on their dissimilarity indices within their respective coordinates.

The five DAMD primers amplified a total of 46 alleles (Table 4). The amplified alleles from each DAMD ranged from 6 to 11, with a mean value of 10.1667. Polymorphic information content values ranged from 0.6519–0.8887 with an average of 0.8268. The DAMD primers including DAMD17R, DAMD13, DAMD16, DAMD1F and DAMD25 were polymorphic. The genetic diversity ranged from 0.6933–0.8978 with a mean value of 0.8430, while the major allele frequency spanned between 0.1333 and 0.4667, with a mean of 0.2556. Detected allelic counts and frequencies from each of the DAMD loci were respectively 1–7 and 0.0667–0.4667 (Additional file 7: Table S5). The genetic diversity in EbPe-2 was the highest, with Ne, H, and I values of 1.9501, 0.4872 and 0.6803, respectively (Table 5). Contrarily, the genetic diversity in EbPe-4 was the lowest, with Ne, H, and I values of 1.0000, 0.0000 and 0.0000, respectively. The genetic diversity values of these accessions were ranked as EbPe-2 > EbPe-6 > CrPe-7 > CrPe-2 > (CrPe-1, EbPe-3) > EbPe-1 > CrPe-3 > EbPe-5 > (CrPe-6, CrPe-8) > EbPe-7 from high to low. The mean values of Ne, H and I obtained from the accessions collected within Cross River State were 1.4015, 0.2693 and 0.4333, respectively, while those from Ebonyi had 1.4520, 0.2776 and 0.4265, respectively. The overall mean values of Ne, H and I detected in the accessions from the two States using DAMD were respectively 1.4268, 0.4081 and 0.6466.

**Table 4:** Allele frequency, number of alleles, gene and polymorphic information content of directed amplified minisatellite DNA

Marker	Major allele frequency	No. of obs.	Allele No	Gene diversity	PIC
DAMD17R	0.2000	15.0000	9.0000	0.8711	0.8574
DAMD13	0.1333	15.0000	11.0000	0.8978	0.8887
DAMD16	0.2667	15.0000	11.0000	0.8711	0.8600
DAMD25	0.4667	15.0000	6.0000	0.6933	0.6519
DAMD1F	0.4000	15.0000	9.0000	0.7911	0.7735
Mean	0.2556	15.0000	10.1667	0.8430	0.8268

*PIC* Polymorphic information content

**Table 5:** Analysis of genetic diversity of 15 accessions of *Capsicum annuum* using directed amplified minisatellite DNA

Pepper Accession	Effective no of alleles, Ne	Nei’s genetic diversity, H	Shannon’s information index, I
CrPe-1	1.4188	0.2952	0.4714
CrPe-2	1.7241	0.4200	0.6109
CrPe-3	1.3272	0.2408	0.4050
CrPe-4	1.2195	0.1800	0.3251
CrPe-5	1.1726	0.1472	0.2788
CrPe-6	1.2677	0.2112	0.3669
CrPe-7	1.8142	0.4488	0.6410
CrPe-8	1.2677	0.2112	0.3669
Sub-mean	1.4015	0.2693	0.4333
EbPe-1	1.3676	0.2688	0.4397
EbPe-2	1.9501	0.4872	0.6803
EbPe-3	1.4188	0.2952	0.4714
EbPe-4	1.0000	0.0000	0.0000
EbPe-5	1.3172	0.2408	0.4050
EbPe-6	1.8911	0.4712	0.6641
EbPe-7	1.2195	0.1800	0.3251
Sub-mean	1.4520	0.2776	0.4265
Overall mean	1.4268	0.4081	0.6466

*CrPe* Cross River State pepper accession and *EbPe* Ebonyi State pepper accession

The genetic variation in pepper accessions from Cross River, assessed using DAMD markers revealed that the mean values of Ht, Hs, G_ST_ and Nm were 0.2693, 0.2465, 0.0992 and 6.4983, respectively (Table 6). Values of Ht, Hs, G_ST_ and Nm recorded in the accessions from Ebonyi were 0.2776, 0.2600, 0.0551 and 17.9512, respectively. The overall values of Ht, Hs, G_ST_ and Nm identified in the entire populations were respectively 0.2732, 0.2528, 0.0746 and 6.2042. The G_ST_ value recorded 0.0746 indicating about 7% of the total genetic divergence among the populations and the remaining 93% within the populations. Across the DAMD markers, NPL and PPL ranged from 8 to 14 and 53.33–86.67, respectively. Effective number of alleles, H and I values produced were 1.2914 ± 0.3779–1.4746 ± 0.3140, 0.1720 ± 1.9880–0.3013 ± 0.1229 and 0.2620 ± 0.2835–0.4656 ± 0.1671, respectively (Additional file 8: Table S6).

**Table 6:** Genetic diversity and differentiation in the accessions of *Capsicum annuum* using directed amplified minisatellite DNA markers

Pepper Accession	Ht	Hs	G_ST_	Nm
CrPe-1	0.2952	0.2600	0.1192	3.6932
CrPe-2	0.4200	0.4040	0.0381	12.6250
CrPe-3	0.2408	0.2200	0.0864	5.2885
CrPe-4	0.1800	0.1560	0.1333	3.2500
CrPe-5	0.1472	0.1280	0.1304	3.3333
CrPe-6	0.2112	0.1840	0.1288	3.3824
CrPe-7	0.4488	0.4360	0.0285	17.0312
CrPe-8	0.2112	0.1840	0.1288	3.3824
Sub-mean	0.2693	0.2465	0.0992	6.4983
EbPe-1	0.2688	0.2400	0.1071	4.1667
EbPe-2	0.4872	0.4840	0.0066	75.6250
EbPe-3	0.2952	0.2840	0.0379	12.6786
EbPe-4	0.0000	0.0000	0.0000	0.0000
EbPe-5	0.2408	0.2360	0.0199	24.5833
EbPe-6	0.4712	0.4120	0.1256	3.4797
EbPe-7	0.1800	0.1640	0.0889	5.1250
Sub-mean	0.2776	0.2600	0.0551	17.9512
Overall mean	0.2732	0.2528	0.0746	6.2042
St. Dev	0.0183	0.0174		

*Ht* total gene diversity, *Hs* gene diversity within population, *G*_*ST*_ coefficient of gene differentiation and *Nm* estimate of gene flow from G_ST_ or Gcs. E.g., *Nm* 0.5(1 - G_ST_)/G_ST_, *CrPe* population of pepper from Cross River State, *EbPe* population of pepper from Ebonyi State

### Comparison of SCoT and DAMD data obtained from the accessions of *Capsicum annuum*

Results obtained from the two markers were compared (Additional file 9: Table S7). Based on the dendrograms, five and three major groups were obtained from SCoT and DAMD data, respectively, while five clusters based on PCA were detected in each of the markers. A total of 57 alleles were found using SCoT markers, while 46 alleles were linked to DAMD markers. The mean numbers of alleles identified in SCoT and DAMD markers were 12.0000 and 10.1667, respectively. Mean genetic diversity values were respectively 0.8815 for SCoT and 0.8430 for DAMD markers, while their mean PICs were respectively 0.8709 and 0.8268. The average major allele frequency associated with the SCoT markers was 0.20000, while that of the DAMD markers yielded 0.2556. The total numbers of polymorphic loci were 64 and 56 for SCoT and DAMD, respectively. The percentage polymorphic loci obtained with the SCoT markers ranged from 80.00–95.73% but 53.33–86.67% with the DAMD markers. Mean of Ne was 1.6971 for SCoT markers and 1.4268 for the DAMD markers. The mean value of H obtained with SCoT markers was 0.5936, while DAMD produced 0.4081. The mean of I obtained with the SCoT and DAMD markers were 0.8590 and 0.6466, respectively.

Also, Ht recorded in the SCoT marker data was 0.3936, while DAMD had 0.2732. The value of Hs in SCoT was 0.3482 and DAMD yielded 0.2528. In SCoT marker data matrix, the G_ST_ was 0.1153 (12% of the total genetic divergence among the populations and 88% within the populations) and 0.0746 (7% of the total genetic divergence among the populations and 93% within the populations) in DAMD, while the Nm obtained with the SCoT marker data was 3.8375 and 0.6.2042 for the DAMD markers.

## Discussion

The efficacy of molecular marker systems for characterization and diversity assessment of *C. annuum* is very important for breeding, improvement and conservation purposes. The estimation of genetic diversity among accessions is beneficial for the utilization and maintenance of genetic resources. Genetic estimation helps to widen the genetic base of the cultivars and prevents gene insertion or deletion [62]. Assessing degree of genetic indices in populations of crops within germplasm using easily affordable and high-proof reading molecular markers could be a useful technique for characterization, detection and selection of novel accessions in a plant breeding-based approach for germplasm conservation of viable genetic resources with potential traits [63, 64]. It will also aid in the verification of identified morphological traits for adequate discrimination of genotypes [65]. Many molecular markers have been used to explore the genetic ingenuity of planting materials and to identify significant phenotypic characters associated with them. Molecular markers of different types have been applied in the characterization, mapping and study of genetic diversity of pepper [23–33]. In this study, SCoT and DMAD markers were explored to comparatively assess the genetic diversity of pepper accessions from Cross River and Ebonyi States of Nigeria. This became necessary since it is widely accepted that comparing different markers to study genetic diversity can be more resourceful and informative in the classification and selection of the best resolving markers [66].

Our results revealed that both SCoT and DAMD markers have high degree of discriminating potential on the pepper accessions as shown by the measured genetic diversity parameters (number of alleles per primer, PIC values, percent polymorphism, and polymorphic loci). Each of the markers, SCoT and DAMD resolved the accessions into five and three major groups, respectively using dendrogram and five clusters each based on PCA. The accessions were differently grouped by the two different sets of markers and this is possible due to variation in the accessibility of different genomic loci by the markers. Two major groups were identified in a study involving 149 black pepper germplasms using EST-SSR markers [33], while five major groups were detected in 30 accessions using SCoT markers [31] and this is in agreement with our findings with SCoT markers. Further, two major groups were found in a study involving 11 Chili pepper using AFLP markers [67]. The discrepancy between dendrograms and clusters of the pepper accessions by the two marker approaches could be linked to the different nature of the markers with regards to their regions of coverage in the genome, level of polymorphism and the number of loci involved as earlier reported [68, 69]. This further demonstrates the significance of total number of identifiable loci and degree of coverage in the whole genome to actualize the required genetic relatedness within accessions [68].

On the part of genetic diversity assessment, SCoT markers identified those from Cross River State to be more genetically isolated compared to those from Ebonyi as a result of higher genetic indices inherent in them. On the other hand, DAMD markers classified accessions from Ebonyi to be more diverse than those from Cross River State. It has been reported that H and I are major parameters in genetic diversity studies since H estimates genetic diversity within and between accessions, while I is for genetic diversity within and between populations regardless of the number of accessions accessed [69, 70]. Based on the higher values in the basic parameters for genetic diversity assessment achieved from the markers (SCoT: H = 0.5936, I = 0.8590; DAMD: H = 0.4081, I = 0.6466), SCoT markers were found to be more efficient than DAMD and this further suggests that accessions from Cross River State are more genetically diverse than those obtained from Ebonyi State. In the present study, H and I, which are independent of number of samples being considered [70], are high in the two markers but higher in SCoT. These identified genetic parameters are higher than the ones obtained from a previous report, where H, I and PIC generated from 30 accessions of pepper using six SCoT markers were 0.2550, 0.4030 and 0.2120, respectively [31]. This difference could be linked to various marker techniques assessing different positions of the genome as previously reported in another combined marker-technique involving ISSR and RAPD [71].

The total potential numbers of identified alleles (SCoT: 57 with mean of 12.0000; DAMD: 46 with mean of 10.1667) in this study demonstrated high allelic richness and genetic diversity within the accessions thereby contributing meaningfully to the genetic diversity among the accessions. The number of alleles detected with the SCoT markers is highly comparable to 53 alleles recently reported using six SCoT and six ISSR markers in 30 accessions of pepper [31] but a bit higher than the one identified with DAMD markers in the present study. In addition, the number of alleles generated with SCoT is lower but its allelic mean value is almost in agreement with a previous report involving 151 alleles, with a mean value of 10.0600 in 39 female *Jojoba* (*Simmondsia chinensis* (Link) Schneider) genotypes amplified with 15 SCoT markers were identified [72]. The obtained PIC using SCoT and DAMD markers were very high but higher in SCoT (PIC range: 0.7735–0.9193; mean: 0.8709) than DAMD (PIC range: 0.6519–0.8887; mean: 0.8268). In other genetic studies, molecular markers with PIC values between 0.4800–0.8000, with a mean of 0.7200 or PIC values above 50% were highly informative and polymorphic ones [73–75]. Both markers demonstrated PIC values higher than the previously reported ones in other crops of female *Jojoba* genotypes analyzed with SCoT markers (PIC range: 0.22–0.4800; mean: 0.4000) [72], *Musa acuminata* with DAMD (PIC range: 0.2900–0.4200; mean: 0.3500) [47] and grapevine cultivars with DAMD (PIC range: 0.3800–0.4900; mean: 0.4400) [76]. The observed mean values of PIC in DAMD is less than that of SCoT which shows that the SCoT markers evaluated are more informative than the DAMD ones. These markers exhibited high percentage polymorphisms (80–95.73% for SCoT and 53.33–86.67% for DAMD markers) in the accessions studied as previously reported on 30 accessions of pepper using SCoT (75–100%) and ISSR (70–100%) [31]. In addition, 33–100% polymorphism was detected from 40 landrace chickpea (*Cicer arietinum* L.) genotypes with SCoT and DAMD markers [77]. The percentage polymorphism obtained is generally high for both marker types, with the SCoT markers revealing higher polymorphisms than DAMD, thereby indicating the efficacious nature of the SCoT marker systems used.

High number of alleles from the two marker polymorphic loci (SCoT: 64; DAMD: 56) demonstrated that they contributed meaningfully to the genetic diversity among these accessions of pepper. This is in contrast with previous reports where 160 alleles were identified with EST-SSR markers in 148 accessions of black pepper [33]. Similarly, 75 and 53 high numbers of alleles of detected from 64 and 30 accessions of pepper were respectively high [28, 31]. The identified genetic parameters from the two markers (SCoT: Ne = 1.6971, H = 0.5936 and I = 0.8590; DAMD: Ne = 1.4268, H = 0.4081 and I = 0.6466) are quite high but higher in SCoT, thereby classifying the accessions from Cross River as the most genetically dissimilar compared to those collected from Ebonyi. In the present study, the identified values of these vital genetic indices are higher than the ones obtained from earlier studies on 30 accessions of pepper using SCoT (H = 0.2550, I = 0.4030) and ISSR (H = 0.3060, I = 0.4590) [31]. The obtained population indices in these accessions using SCoT (Ht = 0.3936, Hs = 0.3482, G_ST_ = 0.1153 and Nm = 3.8375) and DAMD (Ht = 0.2732, Hs = 0.2528, G_ST_ = 0.0746 and Nm = 6.2042) were higher than the ones previously reported in black pepper analyzed with EST-SSR [33]., in pepper accessions with EST-SSR and SCoT [31]., and in wild and domesticated populations of ramie amplified with SCoT [46]. The two marker techniques explicitly detected wide genetic diversity within and between the accessions evaluated, with the SCoT markers showing higher potential in discriminating them.

According to Nei [78], G_ST_ is classified as low when its value is < 0.0500, medium when it is 0.0500 < G_ST_ < 0.1500 and high when G_ST_ > 0.1500. The obtained values of G_ST_ in the two marker systems (SCoT = 0.1153; DAMD: =0.0746) indicate that only about 7 and 12% of the total genetic divergence exist and this may be due to source locations of the accessions (external factors), while 93 and 88% were contributed by genotypic variations within the populations possibly due to internal factors. This finding is consistent with the previous reports [79, 80], where G_ST_ values of 0.1700 and 0.3200 were respectively identified but lower than the ones obtained from chili pepper using AFLP (G_ST_ = 0.8600) [67], *Commiphora wightii* (RAPD: G_ST_ = 0.5800) [81], *Torreya jackii* Chun (ISSR: G_ST_ = 0.6300) [82] and *Curculigo latifolia* (SCoT: G_ST_ = 0.4800) [83]. The noted differences could be attributable to the number of samples analyzed and the nature of the plant genome assessed. The overall genetic variation (Ht = 0.2732) obtained in the whole population with the DAMD marker was lower than the one of intra-population genetic diversity (Hs = 0.2528). On the part of SCoT marker, 0.3936 of Ht was higher than Hs generated as 0.3482. It has been known that the Nm is low when Nm < 1, moderate (Nm > 1) and extensive (Nm > 4) in nature [84]. The recorded Nm in this present study indicates extensiveness in SCoT (Nm = 3.8375) and extremely extensive in DAMD (Nm = 6.2042) gene flow among the populations. This also exhibits a great deal of variation and rich genetic diversity within the populations from different locations across Cross River and Ebonyi States of Nigeria and these unique accessions could be utilized in further hybridization processes in breeding program. This high genetic differentiation within populations/accessions is possibly linked to outcrossing pollination process and exchange of genetic materials as reported in *Begonia* species involving microsatellite markers [85]. In population genetics, the estimation of gene flow with respect to the number of individual genes involves migration of one population to the other and per generation. It induces population differentiation and it is possible to prevent substantial differentiation in a population as a result of genetic drift if the gene flow is greater than 1 (Nm > 1) [86, 87]. Compared to the outcome of this present study, lower values of G_ST_ and Nm have been reported in other crop species including *Rhamnus persicifolia* Moris and *Lilium cernuum* with SSR markers [88, 89].

## Conclusion

Our research revealed that both SCoT and DAMD markers demonstrated a high discriminatory role in the dissection of genetic diversity within the accessions of *C. annuum* from both Ebonyi and Cross River States. However, SCoT markers were found more discriminatory and informative than DAMD markers following the higher genetic diversity indices inherent in SCoT, thereby classifying the accessions from Cross River State as more genetically diverse than those from Ebonyi. The SCoT markers generally were more efficacious by demonstrating higher parameters of all the genetic diversity indices deployed than DAMD markers, except major allele frequency, allele count, allele frequency and gene flow, which may not be good indicators when accessing genetic diversity in *C. annuum* species. Selection from the accessions of the unique clusters and genetic indicators as revealed by the polymorphic SCoT markers could serve as stocks in a hybridization process to achieve superior hybrids capable of withstanding genetic barriers, environmental stressors and novel gene exchange in breeding programs of *C. annuum*.

## Supplementary information

**Additional file 1: Table S1.** Different sample name and sampling locations of the accessions of *Capsicum annuum* studied.

**Additional file 2: Table S2.** List of start codon targeted and directed amplified minisatellite DNA primer sequences used in this study.

**Additional file 3: Figure S1.** Principal component analysis of 15 pepper accessions amplified with start codon targeted markers.

**Additional file 4: Table S3.** Allelic scores, count and frequency revealed by start codon targeted markers.

**Additional file 5: Table S4.** Genetic diversity within Cross River and Ebonyi States *Capsicum annuum* species accessions using start codon targeted markers.

**Additional file 6: Figure S2.** Principal component analysis of 15 accessions of pepper amplified with directed amplified minisatellite DNA markers.

**Additional file 7: Table S5.** Allelic score, count and frequency of directed amplified minisatellite DNA markers.

**Additional file 8: Table S6.** Genetic diversity within accessions of pepper collected Cross River and Ebonyi States and amplified using directed amplified minisatellite DNA markers.

**Additional file 9: Table S7.** Summary of comparisons of start codon targeted and directed amplified minisatellite DNA marker data in pepper accessions.

## Data Availability

The dataset(s) supporting the conclusions of this article is (are) included within the article (and its additional file(s)).

## Abbreviations

AFLP: Amplified fragment length polymorphism
CTAB: Cetyltrimethylammonium bromide
DAMD: Directed amplified minisatellite DNA
DEPC: Diethylpyrocarbonate
EB: Extraction buffer
EDTA: Ethylenediamme tetraacetic acid
EST: Expressed sequence tag
G_ST_: Coefficient of differentiation
H: Nei’s genetic diversity
Hs: Genetic diversity within the population
Ht: Total genetic diversity
I: Shannon’s information index
ISSR: Inter-simple sequence repeat
LGA: Local Government Authority
Ne: Effective number of alleles
Nm: Estimate of gene flow
NPL: Number of polymorphic loci’
PCA: Principal component analysis
PCR: Polymerase chain reaction
PIC: Polymorphic information content
PPL: Percentage polymorphic loci
RAPD: Random amplified polymorphic DNA
RFLP: Restriction fragment length polymorphism
SCoT: Start codon targeted
SNP: Single nucleotide polymorphism
SSR: Simple sequence repeat
UPGMA: Unweighted Pair Group Method with Arithmetic Mean
