# Supplementary material for: Genetic diversity and structure of Capsicum annuum as revealed by start codon targeted and directed amplified minisatellite DNA markers
Source: Hereditas. 2019 Oct 16;156:32. doi: 10.1186/s41065-019-0108-6 (PMC6796447; doi:10.1186/s41065-019-0108-6)
Supplement: Supplementary file 1 — Additional file 1: Table S1. Different sample name and sampling locations of the accessions of Capsicum annuum studied. [file 41065_2019_108_MOESM1_ESM.doc]

**Title: Genetic diversity and structure of *Capsicum annuum* as revealed by Start Codon Targeted and Directed Amplified Minisatellite DNA markers**

**Journal name: Hereditas**

**Author names: David O. Igwe^1,2,3^*, Celestine A. Afiukwa^1,2^, ^3^George Acquaah, ^3^George N. Ude**

**Affiliation and e-mail address of the corresponding author:** ^1^Department of Biotechnology, Faculty of Science, Ebonyi State University, 053, Nigeria; ^2^Biotechnology and Research Development Centre, Ebonyi State University, 053, Ebonyi State, Nigeria; ^3^Department of Natural Sciences, Bowie State University, 14000 Jericho Park Road, Bowie, MD 20715, USA; *****Corresponding author’s contact: digwe@bowiestate.edu; Cell phone number: (443) 741-0645

Additional file 1: Table S1. Different sample name and sampling locations of the accessions of *Capsicum annuum* studied

| **Sample name** | **State** | **Location** | **LGA** |
| --- | --- | --- | --- |
| CrPe-1 | Cross River | Bendi | Obanliku |
| CrPe-2 | Cross River | Iwuru | Biase |
| CrPe-3 | Cross River | Okonde | Boki |
| CrPe-4 | Cross River | Ugeb | Yarkur |
| CrPe-5 | Cross River | Ovunom | Obubura |
| CrPe-6 | Cross River | Awi | Akamkpa |
| CrPe-7 | Cross River | Okuni | Ikom |
| CrPe-8 | Cross River | Ekorinim 2 | Calabar Municipality |
| EbPe-1 | Ebonyi | Amanugwu Edda | Afikpo South |
| EbPe-2 | Ebonyi | Ntezi Aba | Abakaliki |
| EbPe-3 | Ebonyi | Okposi | Ohozara |
| EbPe-4 | Ebonyi | Ezzamgbo | Ohaukwu |
| EbPe-5 | Ebonyi | Ndubia | Izzi |
| EbPe-6 | Ebonyi | Eziulo | Ishielu |
| EbPe-7 | Ebonyi | Amauro Ngbom | Afikpo North |

LGA= Local government area, EbPe = pepper accessions from Ebonyi State, and CrPe = pepper accessions from Cross River.
