# Supplementary material for: Genetic diversity and structure of Capsicum annuum as revealed by start codon targeted and directed amplified minisatellite DNA markers
Source: Hereditas. 2019 Oct 16;156:32. doi: 10.1186/s41065-019-0108-6 (PMC6796447; doi:10.1186/s41065-019-0108-6)
Supplement: Supplementary file 2 — Additional file 2: Table S2. List of start codon targeted and directed amplified minisatellite DNA primer sequences used in this study. [file 41065_2019_108_MOESM2_ESM.doc]

**Title: Genetic diversity and structure of *Capsicum annuum* as revealed by Start Codon Targeted and Directed Amplified Minisatellite DNA markers**

**Journal name: Hereditas**

**Author names: David O. Igwe^1,2,3^*, Celestine A. Afiukwa^1,2^, ^3^George Acquaah, ^3^George N. Ude**

**Affiliation and e-mail address of the corresponding author:** ^1^Department of Biotechnology, Faculty of Science, Ebonyi State University, 053, Nigeria; ^2^Biotechnology and Research Development Centre, Ebonyi State University, 053, Ebonyi State, Nigeria; ^3^Department of Natural Sciences, Bowie State University, 14000 Jericho Park Road, Bowie, MD 20715, USA; *****Corresponding author’s contact: digwe@bowiestate.edu; Cell phone number: (443) 741-0645

Additional file 2: Table S2. List of start codon targeted and directed amplified minisatellite DNA primer sequences used in this study

| **Primer name** | **Primer sequence (5’-3’)** | **% GC** | **Annealing temperature (%)** |
| --- | --- | --- | --- |
| **SCoT** |  |  |  |
| 2 | CAACAATGGCTACCACCC | 55 | 48 |
| 13 | ACGACATGGCGACCATCG | 61 | 48 |
| 16 | ACCATGGCTACCACCGAC | 61 | 48 |
| 20 | ACCATGGCTACCACCGCG | 66 | 48 |
| 22 | AACCATGGCTACCACCAC | 55 | 48 |
| 24 | CACCATGGCTACCACCAT | 50 | 48 |
| 28 | CCATGGCTACCACCGCCA | 66 | 48 |
| 33 | CCATGGCTACCACCGCAG | 61 | 50 |
| 35 | CATGGCTACCACCGGCCC | 72 | 48 |
| 36 | GCAACAATGGCTACCACC | 55 | 48 |
| **DAMD** |  | | |
| URP38F | AAGAGGCATTCTACCACCAC | 50 | 60 |
| URP25F | GATGTGTTCTTGGAGCCTGT | 50 | 60 |
| URP17R | AATGTGGGCAAGCTGGTGGT | 55 | 51 |
| URP13R | TACATCGCAAGTGACACAGG | 50 | 54 |
| URP6R | GGCAAGCTGGTGGGAGGTAC | 65 | 43 |
| URP1F | ATCAAGGTCCGAGACAACC | 55 | 44 |
| 14C2 | GGCAGGATTGAAGC | 64 | 52 |
| 33.6 | AGGGCTGGAGG | 50 | 52 |
| HVA | AGGATGGAAAGGAGGC | 55 | 46 |
| M-13 | GAGGGTGGCGGTCT | 50 | 54 |

**%** GC= Percentage of Guanine-Cytosine
