# Supplementary material for: Genetic diversity and structure of Capsicum annuum as revealed by start codon targeted and directed amplified minisatellite DNA markers
Source: Hereditas. 2019 Oct 16;156:32. doi: 10.1186/s41065-019-0108-6 (PMC6796447; doi:10.1186/s41065-019-0108-6)
Supplement: Supplementary file 3 — Additional file 3: Figure S1. Principal component analysis of 15 pepper accessions amplified with start codon targeted markers. [file 41065_2019_108_MOESM3_ESM.doc]

**Title: Genetic diversity and structure of *Capsicum annuum* as revealed by Start Codon Targeted and Directed Amplified Minisatellite DNA markers**

**Journal name: Hereditas**

**Author names: David O. Igwe^1,2,3^*, Celestine A. Afiukwa^1,2^, ^3^George Acquaah, ^3^George N. Ude**

**Affiliation and e-mail address of the corresponding author:** ^1^Department of Biotechnology, Faculty of Science, Ebonyi State University, 053, Nigeria; ^2^Biotechnology and Research Development Centre, Ebonyi State University, 053, Ebonyi State, Nigeria; ^3^Department of Natural Sciences, Bowie State University, 14000 Jericho Park Road, Bowie, MD 20715, USA; *****Corresponding author’s contact: digwe@bowiestate.edu; Cell phone number: (443) 741-0645


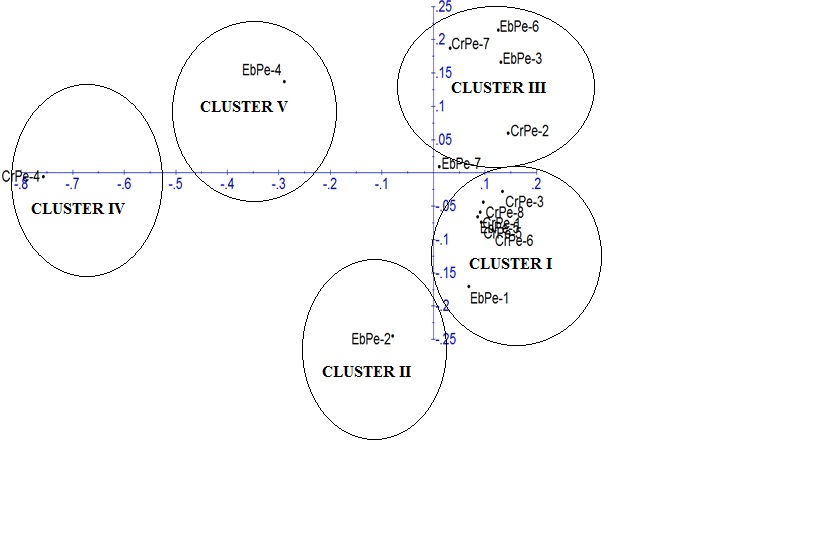


**Additional file 3: Fig. S1** Principal component analysis of 15 pepper accessions amplified with start codon targeted markers
