# Supplementary material for: Genetic diversity and structure of Capsicum annuum as revealed by start codon targeted and directed amplified minisatellite DNA markers
Source: Hereditas. 2019 Oct 16;156:32. doi: 10.1186/s41065-019-0108-6 (PMC6796447; doi:10.1186/s41065-019-0108-6)
Supplement: Supplementary file 4 — Additional file 4: Table S3. Allelic scores, count and frequency revealed by start codon targeted markers. [file 41065_2019_108_MOESM4_ESM.doc]

**Title: Genetic diversity and structure of *Capsicum annuum* as revealed by Start Codon Targeted and Directed Amplified Minisatellite DNA markers**

**Journal name: Hereditas**

**Author names: David O. Igwe^1,2,3^*, Celestine A. Afiukwa^1,2^, ^3^George Acquaah, ^3^George N. Ude**

**Affiliation and e-mail address of the corresponding author:** ^1^Department of Biotechnology, Faculty of Science, Ebonyi State University, 053, Nigeria; ^2^Biotechnology and Research Development Centre, Ebonyi State University, 053, Ebonyi State, Nigeria; ^3^Department of Natural Sciences, Bowie State University, 14000 Jericho Park Road, Bowie, MD 20715, USA; *****Corresponding author’s contact: digwe@bowiestate.edu; Cell phone number: (443) 741-0645

Additional file 4: Table S3. Allelic scores, count and frequency revealed by start codon targeted markers

| **Marker** | **Allele score** | **Allele count** | **Allele frequency** |
| --- | --- | --- | --- |
| SCoT13 | 0/0/0/0/0/0/0/0/0/0/0/0/0/0/0 | 2 | 0.1333 |
| SCoT13 | 0/0/1/0/0/1/0/1/1/1/0/1/0/0/1 | 1 | 0.0667 |
| SCoT13 | 0/0/1/0/0/1/1/1/0/0/1/1/1/0/0 | 1 | 0.0667 |
| SCoT13 | 0/0/1/0/0/1/1/1/1/0/1/1/1/1/1 | 1 | 0.0667 |
| SCoT13 | 0/0/1/0/0/1/1/1/1/1/0/0/0/1/1 | 1 | 0.0667 |
| SCoT13 | 0/0/1/0/1/1/1/1/0/0/1/0/1/0/0 | 1 | 0.0667 |
| SCoT13 | 0/0/1/0/1/1/1/1/1/0/1/1/0/0/1 | 1 | 0.0667 |
| SCoT13 | 0/0/1/1/0/1/0/1/0/0/1/0/0/0/1 | 1 | 0.0667 |
| SCoT13 | 1/0/1/0/0/1/1/1/0/0/1/0/1/0/0 | 1 | 0.0667 |
| SCoT13 | 1/0/1/0/1/1/0/1/0/0/1/1/0/0/1 | 1 | 0.0667 |
| SCoT13 | 1/0/1/0/1/1/0/1/0/1/1/1/0/0/1 | 1 | 0.0667 |
| SCoT13 | 1/0/1/0/1/1/0/1/0/1/1/1/0/1/1 | 1 | 0.0667 |
| SCoT13 | 1/0/1/0/1/1/1/1/0/0/1/1/0/1/1 | 1 | 0.0667 |
| SCoT13 | 1/1/0/0/0/0/0/1/0/0/1/1/0/0/1 | 1 | 0.0667 |
| SCoT28 | 0/0/0/0/0/0/0/0/0/0/0/0 | 2 | 0.1333 |
| SCoT28 | 0/0/0/0/0/0/0/0/0/0/0/1 | 1 | 0.0667 |
| SCoT28 | 0/0/0/0/0/0/0/0/1/0/0/1 | 1 | 0.0667 |
| SCoT28 | 0/0/0/0/0/0/1/0/1/0/0/1 | 1 | 0.0667 |
| SCoT28 | 0/0/0/0/0/1/0/0/0/1/0/1 | 1 | 0.0667 |
| SCoT28 | 0/1/0/0/1/0/0/0/1/0/0/1 | 1 | 0.0667 |
| SCoT28 | 1/0/0/0/0/0/0/0/1/0/0/1 | 2 | 0.1333 |
| SCoT28 | 1/0/0/0/0/0/0/0/1/1/1/1 | 1 | 0.0667 |
| SCoT28 | 1/0/0/0/0/0/1/0/1/0/0/1 | 1 | 0.0667 |
| SCoT28 | 1/0/0/0/0/1/0/0/1/0/0/1 | 1 | 0.0667 |
| SCoT28 | 1/0/0/1/1/1/1/1/1/1/0/1 | 1 | 0.0667 |
| SCoT28 | 1/0/1/0/0/0/0/0/1/0/0/1 | 1 | 0.0667 |
| SCoT28 | 1/0/1/0/1/0/0/0/1/0/0/1 | 1 | 0.0667 |
| SCoT20 | 0/0/0/0/0/0/0/0/0/0/0/0/0 | 2 | 0.1333 |
| SCoT20 | 0/0/0/0/0/1/0/0/0/0/0/0/0 | 1 | 0.0667 |
| SCoT20 | 0/0/1/0/0/0/1/0/0/1/0/0/1 | 1 | 0.0667 |
| SCoT20 | 0/0/1/0/1/1/0/0/0/0/0/0/1 | 1 | 0.0667 |
| SCoT20 | 0/0/1/1/0/0/0/0/0/0/0/1/1 | 1 | 0.0667 |
| SCoT20 | 0/0/1/1/0/0/0/0/0/0/1/1/0 | 1 | 0.0667 |
| SCoT20 | 0/0/1/1/1/1/0/0/0/0/0/0/0 | 1 | 0.0667 |
| SCoT20 | 0/1/1/1/0/0/0/0/0/0/0/0/1 | 1 | 0.0667 |
| SCoT20 | 1/0/1/1/1/0/0/1/0/0/0/1/0 | 1 | 0.0667 |
| SCoT20 | 1/0/1/1/1/1/0/0/0/0/0/0/1 | 1 | 0.0667 |
| SCoT20 | 1/0/1/1/1/1/0/1/0/0/0/0/1 | 1 | 0.0667 |
| SCoT20 | 1/0/1/1/1/1/0/1/0/0/0/1/0 | 1 | 0.0667 |
| SCoT20 | 1/0/1/1/1/1/0/1/1/0/0/0/0 | 1 | 0.0667 |
| SCoT20 | 1/0/1/1/1/1/1/1/0/0/0/1/0 | 1 | 0.0667 |
| SCoT24 | 0/0/0/0/0/0/0/0/0 | 3 | 0.2000 |
| SCoT24 | 0/0/1/0/0/1/1/1/1 | 1 | 0.0667 |
| SCoT24 | 0/0/1/0/1/1/1/1/1 | 1 | 0.0667 |
| SCoT24 | 0/0/1/1/0/1/1/1/1 | 2 | 0.1333 |
| SCoT24 | 0/0/1/1/1/1/1/0/1 | 2 | 0.1333 |
| SCoT24 | 0/0/1/1/1/1/1/1/1 | 5 | 0.3333 |
| SCoT24 | 1/1/0/1/1/1/0/1/1 | 1 | 0.0667 |
| SCoT16 | 0/0/0/0/0/0/0 | 2 | 0.1333 |
| SCoT16 | 0/0/0/0/1/0/1 | 6 | 0.4000 |
| SCoT16 | 0/0/0/0/1/1/1 | 1 | 0.0667 |
| SCoT16 | 0/0/0/1/1/0/1 | 1 | 0.0667 |
| SCoT16 | 0/0/1/0/1/0/1 | 1 | 0.0667 |
| SCoT16 | 0/0/1/0/1/1/1 | 1 | 0.0667 |
| SCoT16 | 0/1/0/0/1/1/1 | 1 | 0.0667 |
| SCoT16 | 1/1/0/0/1/1/1 | 1 | 0.0667 |
| SCoT16 | 1/1/1/0/1/1/1 | 1 | 0.0667 |
