# Supplementary material for: Genetic diversity and structure of Capsicum annuum as revealed by start codon targeted and directed amplified minisatellite DNA markers
Source: Hereditas. 2019 Oct 16;156:32. doi: 10.1186/s41065-019-0108-6 (PMC6796447; doi:10.1186/s41065-019-0108-6)
Supplement: Supplementary file 5 — Additional file 5: Table S4. Genetic diversity within Cross River and Ebonyi States Capsicum annuum species accessions using start codon targeted markers. [file 41065_2019_108_MOESM5_ESM.doc]

**Title: Genetic diversity and structure of *Capsicum annuum* as revealed by Start Codon Targeted and Directed Amplified Minisatellite DNA markers**

**Journal name: Hereditas**

**Author names: David O. Igwe^1,2,3^*, Celestine A. Afiukwa^1,2^, ^3^George Acquaah, ^3^George N. Ude**

**Affiliation and e-mail address of the corresponding author:** ^1^Department of Biotechnology, Faculty of Science, Ebonyi State University, 053, Nigeria; ^2^Biotechnology and Research Development Centre, Ebonyi State University, 053, Ebonyi State, Nigeria; ^3^Department of Natural Sciences, Bowie State University, 14000 Jericho Park Road, Bowie, MD 20715, USA; *****Corresponding author’s contact: digwe@bowiestate.edu; Cell phone number: (443) 741-0645

Additional file 5: Table S4. Genetic diversity within Cross River and Ebonyi States *Capsicum annuum* species accessions using start codon targeted markers

| Marker | NPL | PPL | Ne | H | I |
| --- | --- | --- | --- | --- | --- |
| **SCoT13** | 13 | 95.73 | 1.8026(0.3338) | 0.4160(0.1701) | 0.5831(0.2376) |
| **SCoT28** | 13 | 95.73 | 1.4945(0.2817) | 0.3046(0.1522) | 0.4603(0.2148) |
| **SCoT20** | 13 | 95.73 | 1.6538(0.3503) | 0.3615(0.1743) | 0.5223(0.2393) |
| **SCoT24** | 12 | 80.00 | 1.7575(0.3953) | 0.3887(0.2017) | 0.5431(0.2815) |
| **SCoT16** | 13 | 95.73 | 1.4149(0.2594) | 0.2702(0.1379) | 0.4218(0.1962) |

Standard deviations are in parentheses, NPL=number of polymorphic loci, PPL=percentage polymorphic loci, Ne = Effective number of alleles, H = Nei's gene diversity, I = Shannon's Information index
