# Supplementary material for: Genetic diversity and structure of Capsicum annuum as revealed by start codon targeted and directed amplified minisatellite DNA markers
Source: Hereditas. 2019 Oct 16;156:32. doi: 10.1186/s41065-019-0108-6 (PMC6796447; doi:10.1186/s41065-019-0108-6)
Supplement: Supplementary file 8 — Additional file 8: Table S6. Genetic diversity within accessions of pepper collected Cross River and Ebonyi States and amplified using directed amplified minisatellite DNA markers. [file 41065_2019_108_MOESM8_ESM.doc]

**Title: Genetic diversity and structure of *Capsicum annuum* as revealed by Start Codon Targeted and Directed Amplified Minisatellite DNA markers**

**Journal name: Hereditas**

**Author names: David O. Igwe^1,2,3^*, Celestine A. Afiukwa^1,2^, ^3^George Acquaah, ^3^George N. Ude**

**Affiliation and e-mail address of the corresponding author:** ^1^Department of Biotechnology, Faculty of Science, Ebonyi State University, 053, Nigeria; ^2^Biotechnology and Research Development Centre, Ebonyi State University, 053, Ebonyi State, Nigeria; ^3^Department of Natural Sciences, Bowie State University, 14000 Jericho Park Road, Bowie, MD 20715, USA; *****Corresponding author’s contact: digwe@bowiestate.edu; Cell phone number: (443) 741-0645

Additional file 8: Table S6. Genetic diversity within accessions of pepper collected Cross River and Ebonyi States and amplified using directed amplified minisatellite DNA markers

| **Marker** | **NPL** | **PPL** | **Ne** | **H** | **I** |
| --- | --- | --- | --- | --- | --- |
| DAMD17R | 12 | 80 | 1.4746(0.3140) | 0.2880(0.1725) | 0.4326(0.2459) |
|
| DAMD13 | 13 | 80.33 | 1.4501(0.3559) | 0.2720(0.1705) | 0.4183(0.2272) |
|
| DAMD16 | 14 | 86.67 | 1.4699(0.2403) | 0.3013(0.1229) | 0.4656(0.1671) |
|
| DAMD25 | 8 | 53.33 | 1.2914(0.3779) | 0.1720(0.1988) | 0.2620(0.2835) |
|
| DAMD1F | 9 | 60 | 1.4002(0.3974) | 0.2307(0.2112) | 0.3405(0.3021) |
|

Standard deviations are in parentheses, NPL=number of polymorphic loci, PPL=percentage polymorphic loci, Ne = Effective number of alleles, H = Nei's gene diversity, I = Shannon's Information index
