# Supplementary material for: Genetic diversity and structure of Capsicum annuum as revealed by start codon targeted and directed amplified minisatellite DNA markers
Source: Hereditas. 2019 Oct 16;156:32. doi: 10.1186/s41065-019-0108-6 (PMC6796447; doi:10.1186/s41065-019-0108-6)
Supplement: Supplementary file 9 — Additional file 9: Table S7. Summary of comparisons of start codon targeted and directed amplified minisatellite DNA marker data in pepper accessions. [file 41065_2019_108_MOESM9_ESM.doc]

**Title: Genetic diversity and structure of *Capsicum annuum* as revealed by Start Codon Targeted and Directed Amplified Minisatellite DNA markers**

**Journal name: Hereditas**

**Author names: David O. Igwe^1,2,3^*, Celestine A. Afiukwa^1,2^, ^3^George Acquaah, ^3^George N. Ude**

**Affiliation and e-mail address of the corresponding author:** ^1^Department of Biotechnology, Faculty of Science, Ebonyi State University, 053, Nigeria; ^2^Biotechnology and Research Development Centre, Ebonyi State University, 053, Ebonyi State, Nigeria; ^3^Department of Natural Sciences, Bowie State University, 14000 Jericho Park Road, Bowie, MD 20715, USA; *****Corresponding author’s contact: digwe@bowiestate.edu; Cell phone number: (443) 741-0645

Supplementary Table S7. Summary of comparisons of start codon targeted and directed amplified minisatellite DNA marker data in pepper accessions

| **Parameters** | **Markers** | |
| --- | --- | --- |
|  | **SCoT** | **DAMD** |
| No of clusters by dendrogram | 5.000 | 3.000 |
| No of clusters by PCA  No of observation | 5.000  15.000 | 5.000  15.000 |
| Total no of alleles | 57.000 | 46.000 |
| Mean no of alleles | 12.000 | 10.1667 |
| Mean gene diversity | 0.8815 | 0.8430 |
| Mean PIC  Mean major allele frequency | 0.8709  0.2000 | 0.8268  0.2556 |
| Total No of polymorphic loci (NPL) | 64.000 | 56.000 |
| Range of percentage polymorphic loci (PPL) | 80.00-95.73% | 53.33-86.67% |
| Mean effective alleles (Ne) | 1.6971 | 1.4268 |
| Mean Nei's gene diversity (H) | 0.5936 | 0.4081 |
| Mean Shannon's information index (I)  Mean total gene diversity (Ht)  Mean gene diversity within population (Hs)  Mean coefficient of gene differentiation (Gst)  Mean estimate of gene flow (Nm) | 0.8590  0.3936  0.2528  0.0746  3.8375 | 0.6466  0.2732  0.3482  0.1153  6.2042 |

PIC=Polymorphic information content; PCA=Principal component analysis; SCoT=Start codon targeted; and DAMD=Directed amplified minisatellite DNA
